# Study of Properties of Composite Cementitious Materials with Sulfoaluminate Cement and Solid Waste Based on Compaction Forming Process

**DOI:** 10.3390/ma18092076

**Published:** 2025-05-01

**Authors:** Zhiyao Ma, Xujiang Wang, Mushen Yu, Shouyan Chen, Jiwen Liu, Jingwei Li, Jianyong Wang, Hao Sun, Yanpeng Mao, Zhijuan Hu, Wenlong Wang

**Affiliations:** 1 National Engineering Laboratory for Reducing Emissions from Coal Combustion, Shandong University, Jinan 250061, China; 202214488@mail.sdu.edu.cn (Z.M.); chenshouyan@sdu.edu.cn (S.C.); sdgsldljxm@163.com (J.L.); ljw@sdu.edu.cn (J.L.); maoyanpeng@sdu.edu.cn (Y.M.); huzhijuan@sdu.edu.cn (Z.H.); wwenlong@sdu.edu.cn (W.W.); 2 Shenzhen Research Institute, Shandong University, Shenzhen 518000, China; 3 Shandong Road & Bridge Construction Group Co., Ltd., Jinan 250014, China; sdumarch@163.com; 4 PowerChina Sepco1 Electric Power Construction Co., Ltd., Jinan 250102, China; wjy6240@sepco1.com

**Keywords:** industrial solid waste, composite cementitious materials, compaction forming, mechanical properties, porosity

## Abstract

The traditional cement compaction process boasts notable advantages such as high strength, durability, and aesthetic appeal. However, compaction forming technology for cement products results in high carbon emissions. Consequently, it is imperative to develop low-carbon strategies for these products. This study investigates the modification of solid waste-based low-carbon sulfoaluminate cementitious material (SSCM) using mineral powder (MP) and steel slag micro powder (SSMP) under compaction forming technology. The results indicate that higher compaction pressure leads to higher early-stage strength, while the later-stage strength is primarily influenced by the degree of hydration. At a compaction pressure of 40 MPa, the one-day compressive strength of the material exceeded 70 MPa, representing a 48.31% increase compared to the control group. Under compaction forming, the hydration reaction rate decreased, but the compaction process significantly reduced porosity. Moreover, higher pressure correlated with a reduction in the proportion of harmful pores. Incorporating 25% MP and 20% SSMP increased the compressive strength by 10.66% to 113.5 MPa. According to orthogonal experimental results, the optimal molding conditions entail a 20% MP content, a 20% SSMP content, and a molding pressure of 40 MPa. The research findings can serve as a theoretical foundation for the widespread application of SSCM and compaction forming technology in practical engineering.

## 1. Introduction

Compaction forming technology is widely used to manufacture high-performance cementitious composites, extensively employed in structural components and infrastructure systems [1]. Compacted cement products demonstrate remarkable advantages in practical applications, characterized by their high strength, superior durability, shortened molding cycles, and excellent aesthetic quality. They stand as potential replacements for natural marble and traditional artificial stones. Annually, the global production of compacted cement products exceeds 4.1 billion tons. At a compaction pressure of 350 MPa and a water-to-cement ratio of 0.21, a hardened cementitious paste with a compressive strength of 645 MPa can be obtained [2,3]. While the compaction method is widely used for manufacturing cement-based materials, there remains limited understanding of how key process parameters influence the final product’s performance. The primary reason lies in key parameters like the water-to-cement ratio, which is routinely adjusted during actual production in response to variables such as temperature, humidity, and raw material properties. Furthermore, the compaction technology for cement products poses several environmental concerns. Primarily, cement-based materials based on compaction technology typically contain high cement content (65–85%) [4], which inevitably leads to significantly increased carbon emissions. This issue is further exacerbated by the predominant use of ordinary Portland cement (OPC) in this process, as OPC exhibits substantially higher carbon emissions (0.83 kg CO_2_/kg) compared to aggregates (0.01 kg CO_2_/kg) [5]. Additionally, the requirement for exceptionally high forming pressures unavoidably results in greater energy consumption, thereby compounding the environmental impacts associated with these products. The global carbon emission issue remains a pressing challenge. Therefore, minimizing carbon emissions from cement production is imperative.

To address the issue of high carbon emissions, two main aspects can be undertaken. Firstly, seeking alternatives to traditional cementitious materials is crucial [6,7]. Compared to OPC, the clinkering temperature of calcium sulfoaluminate cement (CSA) is 100–200 °C lower, consequently leading to lower CO_2_ emissions during its preparation [8,9]. CSA possesses excellent characteristics such as shorter setting time, higher early strength, erosion resistance, and slight expansion, making it extensively utilized in specialized engineering projects [10,11]. In recent years, several studies have successfully utilized industrial solid waste to produce high-performance CSA [12,13,14]. Research has confirmed that utilizing solid waste to produce CSA cement can reduce carbon emissions to below 450 kgCO_2_/tcl [15,16]. Utilizing industrial solid waste to produce CSA offers the opportunity for further reductions in raw material costs and carbon emissions.

On the other hand, industrial waste materials can be utilized as mineral admixtures in concrete to reduce cement consumption, thereby lowering costs and decreasing carbon emissions in cement production [17]. By finely grinding these industrial solid waste materials and subsequently incorporating them into cementitious materials as mineral admixtures, it serves the dual purpose of enhancing various properties of cementitious materials, such as mitigating the heat of hydration and improving mechanical performance, while also facilitating the high-value utilization of industrial solid waste [18]. The influence of different mineral admixtures, including flying ash [19,20,21], silica fume [22], carbonate minerals [23,24,25], and metakaolin [26], on cement hydration has been widely studied. Mineral powder (MP) and steel slag powder are industrial solid wastes generated by the iron and steel industry during the smelting of pig iron, with their main chemical components including CaO, SiO_2_, and Al_2_O_3_, among others [27,28]. Previous studies have shown that mineral powder and steel slag exhibit good synergy with cement and they are thus widely used as mineral admixtures in cement [29].

While there is substantial research on the use of industrial solid waste as mineral admixtures in cement hydration, the study of the cementitious properties of multi-component systems involving the combination of CSA and industrial solid waste is still notably lacking. It is essential to determine the optimal blending ratios and forming parameters to achieve a significant reduction in carbon emissions in the static pressing process. This study primarily investigated the modification of SSCM using industrial solid waste (mineral powder and steel slag micro-powder) and assessed the impact of forming pressure and mineral admixture content on the performance of SSCM materials under static pressing. A series of laboratory tests were conducted to evaluate the physical and mechanical properties of SSCM. Additionally, X-ray diffraction (XRD), mercury intrusion porosimeter (MIP), and thermogravimetric analysis (TGA) analyses were employed to examine the mineral composition, microstructure, and thermal weight loss characteristics of the specimens. The research findings provide a theoretical basis for the widespread application of SSCM and static pressing processes in practical engineering.

## 2. Materials and Methods

### 2.1. Materials

**Figure 1:**
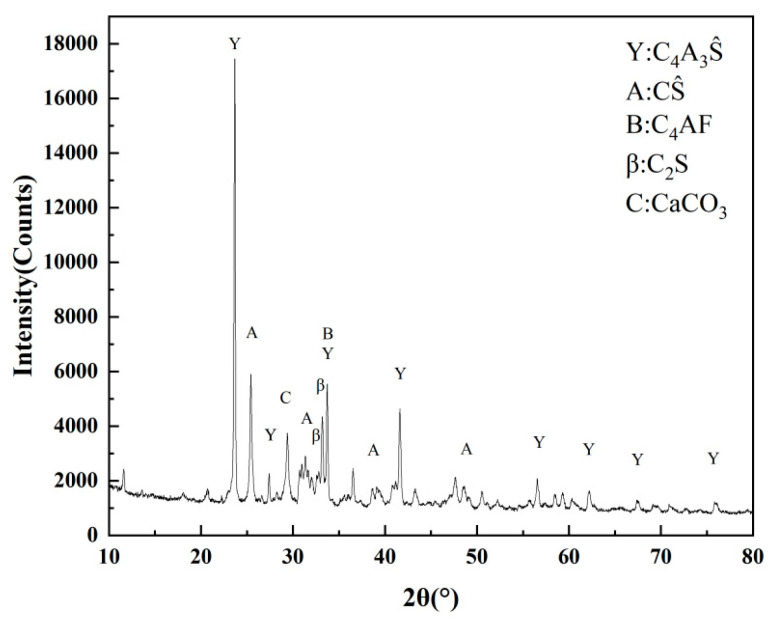
XRD pattern of SSCM.

**Figure 2:**
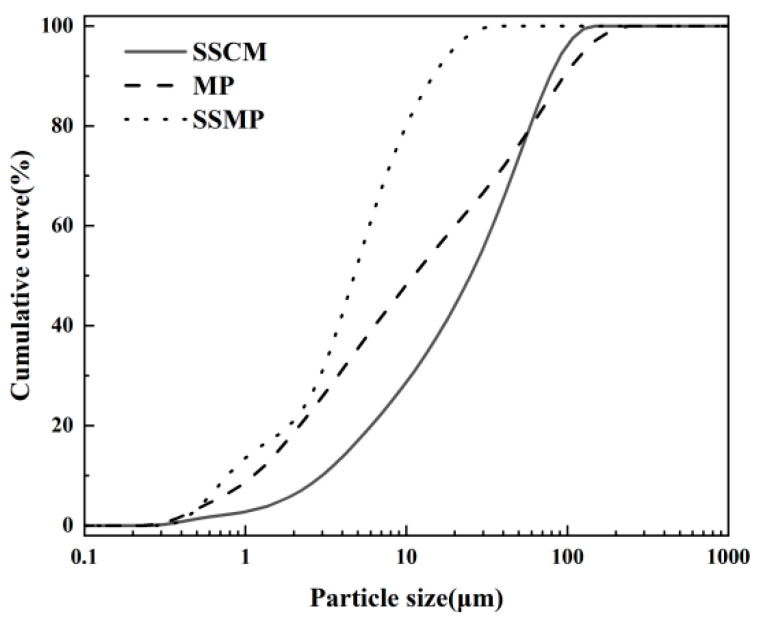
Particle size distribution curve of raw materials.

The cementitious material used in the experiments was a completely solid waste-based sulfoaluminate low-carbon cementitious material (SSCM) [14]. All the raw materials used in its preparation, including steel slag, aluminum ash, limestone tailings, and desulfurization gypsum, were derived from industrial solid waste. Its primary characteristics are presented in Table 1. The mineral composition of the SSCM was determined through XRD analysis, as depicted in Figure 1. The steel slag was sourced from Hebei Yanshan Iron and Steel Group (Tangshan, China). The steel slag was subjected to pre-grinding using a ball mill and then ultrafine grinding using a supersonic steam jet mill to prepare steel slag micro-powder (SSMP). The specific surface area of the SSMP was 710 m^2^/kg. The mineral powder used was commercially available S95-grade mineral powder with a specific surface area of 468 m^2^/kg. The particle size distribution of the raw materials is shown in Figure 2. The chemical compositions of the applied binders were determined by X-ray fluorescence spectrometry (XRF) and are given in Table 2.

### 2.2. Experimental Program

Initially, specimens of SSCM material were prepared under molding pressures of 20 MPa, 30 MPa, and 40 MPa to investigate the influence of pressure on the properties of statically pressed specimens. To illustrate the effect of static pressure on the samples, comparisons were also made with conventional cast-forming. Subsequently, with a fixed molding pressure of 20 MPa, the impact of mineral powder and steel slag micro-powder on the specimen’s properties was studied, as shown in Table 3 for the experimental design ratios. Finally, considering molding pressure, the proportion of SSMP, and the proportion of mineral powder, an orthogonal experimental design was devised.

Orthogonal experimental design L9 with three factors and three levels was employed in this study. Orthogonal experimental design is widely employed in the experimental design of cement-based materials to analyze the effects of multiple factors and their interactions on experimental results [30,31]. The three factors considered were the MP (A), SSMP (B), and forming pressure (C). The specific values for each factor level are presented in Table 4, and the mass dosages are expressed as fractions of the total dry mass. A comprehensive analysis, including visual inspection, range analysis, variance analysis, and overall assessment, was conducted to discuss the results of the orthogonal experiment and determine the optimal mixture ratio.

Following the experimental mixture ratio, the SSCM material, SSMP, and MP were homogeneously mixed for 3 min. Water was added according to a w/c of 0.14 and mixed thoroughly. 15 g of the moist composite binder material was placed into specially designed molds and compacted under the appropriate pressure to produce cubic specimens measuring 20 mm × 20 mm × 20 mm. After forming, the specimens were transferred to a temperature-controlled and humidity-controlled curing chamber maintained at 20 ± 2 °C with a relative humidity of ≥95% for further curing.

### 2.3. Methods

The compressive strength of the 20 mm × 20 mm × 20 mm cubic specimens was determined using a universal mechanical testing machine(WDW-100, Gaosheng Test Instrument Co., Jinan, China). All tests were performed at a constant loading rate of 0.5 N/s. Compressive strength was calculated from the average of six replicate specimens.

The mineral composition and content of the SSCM and hydrated paste were analyzed using XRD (Aeris, Malvern Panalytical, Malverin, UK) equipped with Cu-Kα radiation. Samples were analyzed at 40 kV and 15 mA, using a scanning rate of 0.04°/s.

MIP (AutoPore V 9600, Micromeritics Instrument Corporation, Norcross, GA, USA), with a measurement range of 0–60,000 psi (414 MPa), characterized the pore size distribution of the hardened samples. This instrument can measure pores with diameters of ≥5.0 nm.

TG ( TGA/DSC 1/1600, Mettler Toledo, Greifensee, Switzerland) was employed to measure the mass change of the samples with temperature. About 10 mg of the sample was heated from 30 °C to 600 °C at 10 °C/min under a nitrogen (N2) atmosphere.

## 3. Results and Discussion

### 3.1. Effect of Forming Pressure on the Strength of SSCM

**Figure 3:**
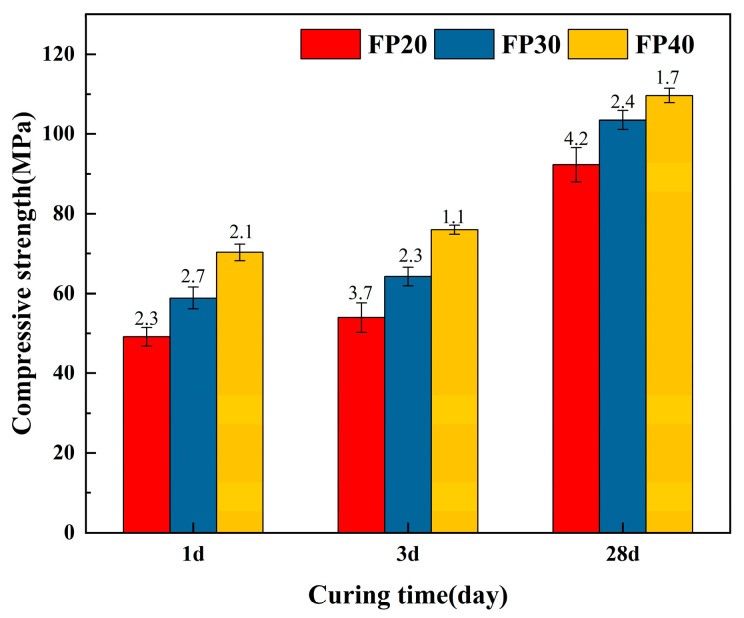
Compressive strength development of the SSCM with different forming pressures.

Figure 3 shows the compressive strength of SSCM at different curing ages under forming pressures of 20 MPa, 30 MPa, and 40 MPa. After hydration for 1 day, the compressive strength of the FP40 group had already exceeded 70 MPa and, with increasing curing time, the material’s strength continuously increased, reaching over 110 MPa at 28 days. This indicated that the SSCM material exhibited high strength both in the early and late stages of hydration under the pressure forming process.

The compressive strength of SSCM consistently increased with higher forming pressure at all curing ages. In the early stage of hydration, the effect of forming pressure on the strength of the samples was significant. After hydration for 1 day, the FP40 group exhibited a 48.31% increase in compressive strength compared to the FP20 group. However, the pressure-induced strength enhancement became less pronounced during later hydration phases. Firstly, the influence of pressure on the mechanical properties of cement-based materials was immediate. During the forming process, it instantaneously reduced and compressed pores, thereby enhancing initial compactness. Consequently, this effect predominated in enhancing compressive strength during the early hydration stage [32,33]. In contrast, hydration reactions progressed gradually with increasing curing age. Due to the dry-mixing process employed in compaction forming, the water-to-cement ratio remained relatively low. This resulted in a comparatively low degree of hydration in early stages, making mechanical properties primarily dependent on forming pressure [34]. As hydration proceeded, the formation of hydration products (AFt and AH_3_) contributed to progressive strength development. Thus, the later-stage compressive strength became predominantly governed by hydration reactions.

With the progression of hydration reactions, at 28 days, the FP40 group’s strength increased by 17.85% compared to the FP20 group, indicating that the later-stage strength growth was primarily due to SSCM hydration. It can be expected that within a considerable range, material strength will continue to increase with higher forming pressure [35]. However, excessively high forming pressure not only results in higher costs but may also damage the internal structure of the compacted material [36].

### 3.2. Effect of Forming Pressure on the Pore Structure of SSCM

**Figure 4:**
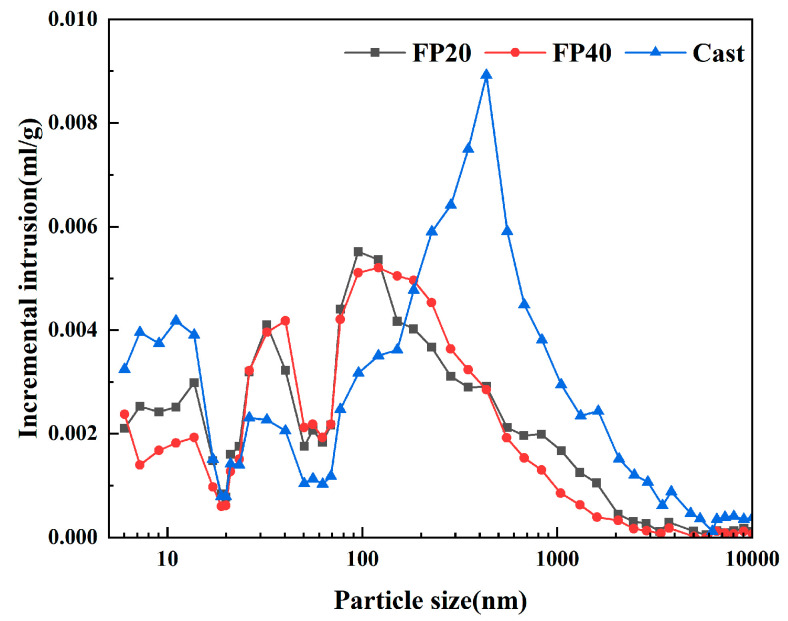
Pore size distributions of SSCM after curing for 28 days.

**Figure 5:**
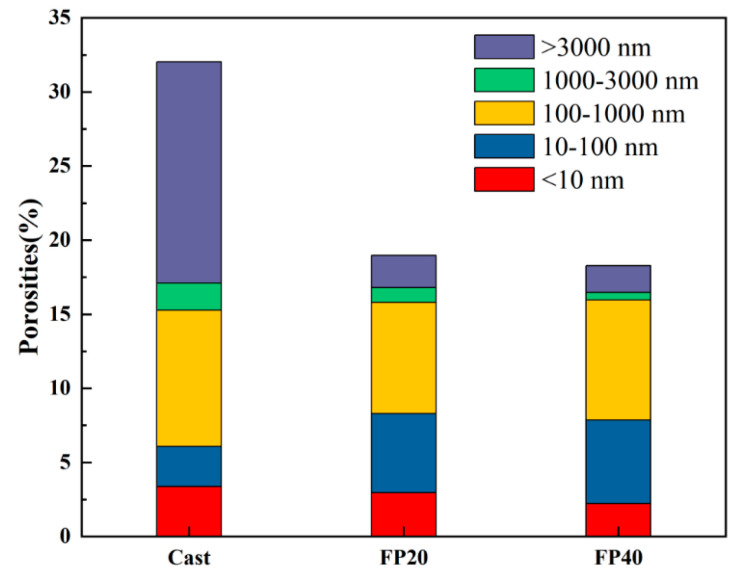
Porosity of materials under different pressures and casting processes.

Figure 4 and Figure 5 present the pore size distribution curves and porosity of the materials at 28 days of hydration under forming pressures of 20 MPa and 40 MPa and a cast-forming process. From the distribution curves, it can be observed that the most probable pore sizes for the SSCM cast, FP20, and FP40 groups were 433 nm, 120 nm, and 183 nm. Comparatively, the most probable pore size was significantly reduced in the static pressure forming process compared to the cast-forming process.

Combining this with Figure 5, it can be seen that the total porosity of FP20 and FP40 was 18.96% and 18.27%, which was much lower than the 32.04% observed for the cast-forming process. This indicated that although static pressure forming reduced the water-to-cement ratio and hydration degree, the material’s porosity was much lower than that of the cast-forming process. The pores in cement can be categorized as gel pores (<10 nm), small capillary pores (10–100 nm), large capillary pores (100–1000 nm), and air holes (>1000 nm). Generally, pores larger than 100 nm are considered to be detrimental and pores larger than 3000 nm are considered to be significantly detrimental to strength development [37]. During the casting process, the relatively high water-to-cement ratio and slow hydration progress resulted in the significant retention of air voids and large capillary pores within the samples, which evidently compromised their mechanical performance [38]. In contrast, compaction forming employed a lower water-to-cement ratio that virtually eliminated air holes, while the applied high pressure simultaneously compressed macro-pores and substantially reduced micro-pores. This was also one of the reasons for the higher strength in the static pressure-formed group [39,40].

The porosity under different forming conditions revealed that the total porosity of the samples showed minimal variation during the later hydration stages. The FP40 specimen exhibited lower porosity than FP20, with a 0.87% reduction in air hole content, primarily attributable to differences in forming pressure during early hydration. As established in previous studies, hydration products predominantly filled capillary and gel pores within the specimens [41]. At 28 days of hydration, minor differences were observed in the proportion of capillary and gel pores among different samples, indicating that forming pressure exerted limited influence on the later-stage hydration process. Consistent with the observed trend in compressive strength, pressure predominantly influenced SSCM during the initial stages, enhancing mechanical properties by improving pore structure.

### 3.3. Effect of Forming Pressure on the Hydration Process of SSCM

**Figure 6:**
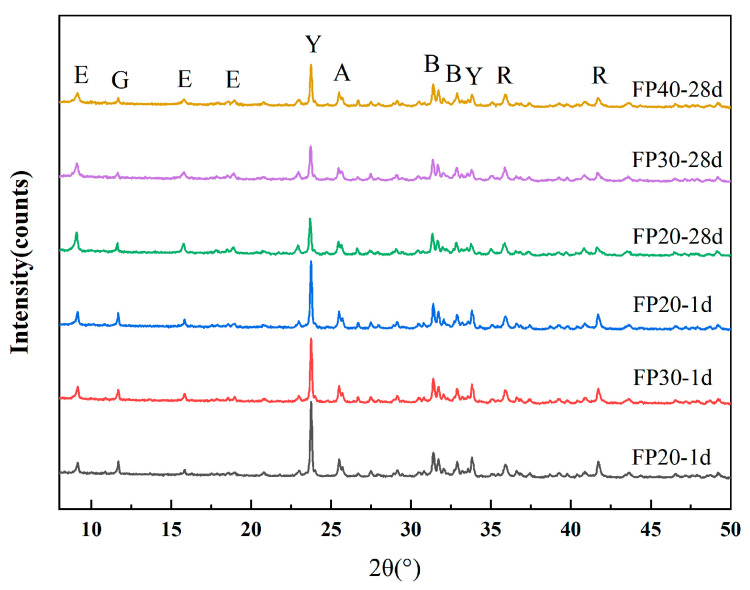
XRD patterns of different samples cured for 1 d and 28 d (E-AFt, G-gypsum, Y-ye’elimite, A-anhydrite, B-belite, R-RO phase).

The mineral composition of SSCM materials at 1 day and 28 days of hydration under different forming pressures is shown in Figure 6 and Table A1. It can be observed that under static pressure forming, the main hydration product was AFt, but the hydration reaction rate was very slow. Even at 28 days, significant amounts of anhydrite and gypsum could still be observed. Under different forming pressures, there was no change in the types of hydration products.

**Figure 7:**
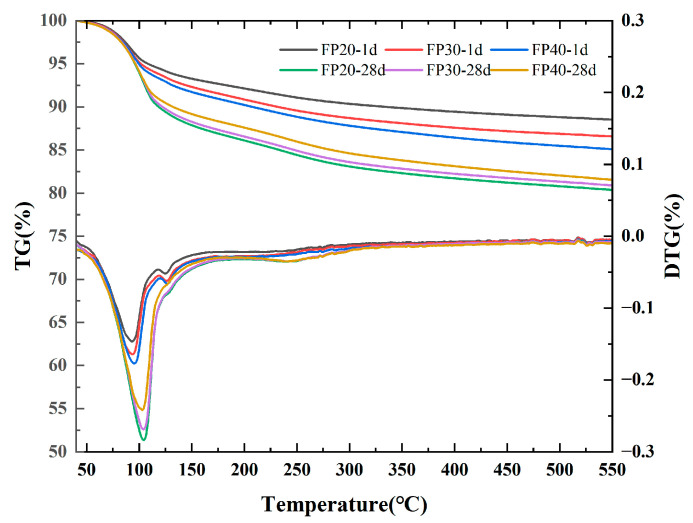
TGA and DTG curves of different samples cured for 1 d and 28 d.

The TG-DTG curves of SSCM at different ages of hydration are shown in Figure 7. After 1 day of hydration, the weight loss under forming pressures of 20 MPa, 30 MPa, and 40 MPa was 10.09%, 10.61%, and 10.71%. After 28 days, the weight loss was 15.49%, 15.23%, and 14.9%, respectively. It could be observed that higher forming pressures resulted in the formation of more AFt at 1 day, but the opposite trend occurred at 28 days. This was different from the results obtained under higher w/c ratios in a previous study [42]. The reason for this difference may be that the initially high compactness promoted tighter particle binding and enhanced the hydration reaction in the early stages. However, in the later stages, excessive compactness hindered water vapor penetration into the interior of the specimens, and the low initial w/c ratio limited the amount of water available for hydration reactions, thereby inhibiting the formation of hydration products. Compared to the cast-forming process, it could be seen that due to the lower w/c ratio, the static pressure-formed materials exhibited a significantly reduced hydration reaction rate in the early stages and a lower overall hydration degree. Therefore, the material’s strength was predominantly governed by the static pressure applied during the forming process, while the pressure exerted partial inhibitory effects on hydration.

### 3.4. Effect of MP and SSMP Content on the Strength of SSCM

**Figure 8:**
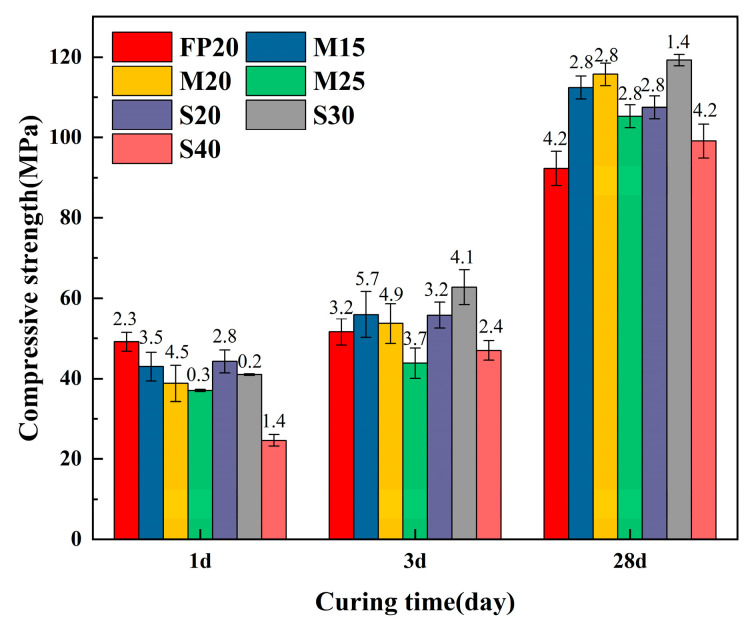
Compressive strength of the material with single admixture.

The influence of varying proportions of MP and SSMP on the compressive strength of SSCM at a molding pressure of 20 MPa is illustrated in Figure 8. After 1 day of hydration, as the proportion of mineral powder increased, the strength gradually decreased. This phenomenon can be attributed to the fact that early strength development in CSA is primarily driven by the rapid hydration of ettringite. The mineral powder replaced some of the SSCM and had minimal involvement in the early hydration reactions. However, as the hydration period progressed, MP became more active due to activation by the hydration products of cement [43]. It should be noted that unlike casting processes, the dry-mixing method renders the particle size distribution inconsequential to the raw materials’ flow characteristics, affecting only their particle packing behavior. This influence becomes further attenuated after pressure forming.

The incorporation of SSMP resulted in a decreasing trend in material strength at 1 day, with a more pronounced decrease observed with higher content. This differed from previous literature findings, attributed to the higher water-to-cement ratio prevalent in casting processes, which accelerated the dissolution of steel slag, consequently elevating the pH of the pore solution and accelerating hydration, thereby enhancing early strength. At 3 days of hydration, the dissolution of SSMP and SSCM material significantly increased, resulting in a rise in pore solution pH and the acceleration of hydration reactions [44]. Consequently, at 3 days of hydration, the strength of the S20 and S30 groups exceeded that of the FP20 group. By 28 days of hydration, with the material undergoing thorough hydration, the addition of 30% SSMP resulted in the greatest strength enhancement, with an increase of 29.25%. When the steel slag powder content was 40%, there was still an improvement in compressive strength compared to the SSCM20 group. However, based on the trend of decreasing strength, it could be inferred that when the steel slag powder content exceeded 40%, there would be a significant decrease in material compressive strength.

**Figure 9:**
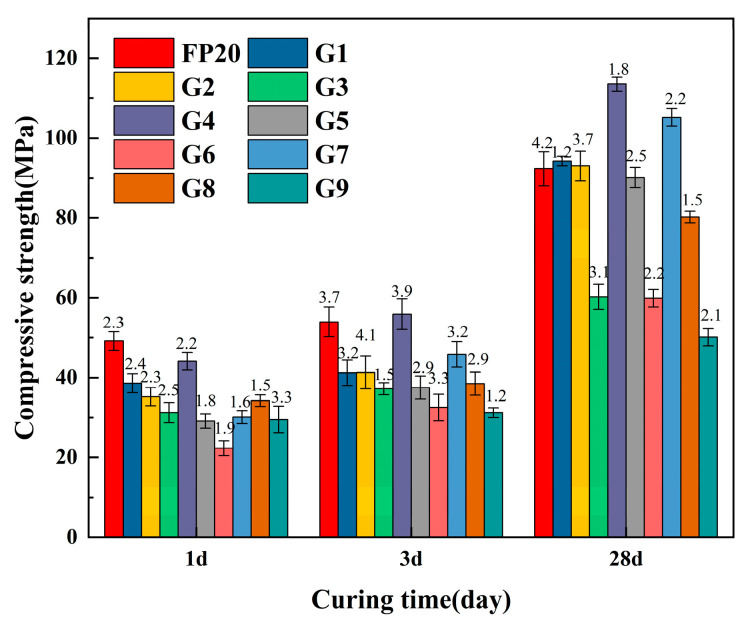
Compressive strength of the material with composite admixture.

The compressive strength of ternary composite cementitious materials composed of different proportions of MP, SSMP, and SSCM materials is illustrated in Figure 9. At 1 day, the strength of groups G1–G9 was lower than that of FP20. This was primarily because MP and SSMP had minimal positive effects during the early hydration stage when the water-to-binder ratio was low, but their dilution effect could compromise material performance. After 28 days, an appropriate combination of additives could enhance material strength. For instance, group G4 exhibited a 13.81% increase in strength, indicating that the synergistic cementitious action of MP and SSMP, at suitable proportions, could improve material performance. However, excessive additive content should be avoided. When the total content reached 55%, the compressive strength of the resulting material decreased by 18.54% relative to the FP20 group. Therefore, the total additive content should be kept below 50%. Comparing G7 with G2 and G8 with G3, it can be observed that maintaining an MP to SSMP ratio of approximately 1:1 maximized the synergistic effect of the materials. Moreover, the combined incorporation of MP and SSMP enabled higher SSCM replacement ratios compared to their individual addition.

### 3.5. Process Optimization of Materials Under Compaction Forming Technology

An analysis of the experimental results based on the orthogonal experimental design is presented in Table 5. The influencing factors in the table are denoted as follows: A (MP content), B (SSMP content), and C (forming pressure). The term “R” represents the range, and a larger range indicates that variations in the levels of this factor had a greater impact on the experimental results. Therefore, a larger range suggests that this factor had a greater influence on the material strength among the three factors.

From Table 5, it is evident that during the molding of SSCM at different pressures, the factors affecting material performance ranked in the following order of significance: B > C > A. It can be observed from the results that the compressive strength initially increased and then decreased with an increase in the dosage of MP, reaching an optimal level at A2. The compressive strength gradually decreased with an increase in the dosage of SSMP, reaching an optimal level at B1. Furthermore, an increase in molding pressure led to a corresponding increase in compressive strength, reaching an optimal level at C3. Regarding the trend of experimental results, it was noted that after the MP dosage reached 20%, further additions significantly affected the compressive strength of the material. Although increasing the dosage of SSMP from 20% to 30% had minimal impact on compressive strength, continued addition resulted in a substantial decrease in strength. Additionally, the increase in forming pressure proportionally enhanced strength, with higher molding pressure resulting in the greater compressive strength of the material.

In summary, the optimal experimental conditions corresponding to the material’s peak performance were identified as A2B1C3. Although the optimal conditions were not explicitly included in the experimental design, the orthogonal experiment did incorporate the condition of A2B1C2 (P4 group), which yielded a compressive strength very close to the highest value observed in the designed experiments. Previous analyses clearly demonstrate that higher forming pressure enhances the compressive strength of samples at all curing ages. As demonstrated in Section 3.1 and Section 3.2, higher forming pressure significantly improved the compressive strength of samples across all curing ages. It is foreseeable that the optimal experimental conditions for compressive strength are A2B1C3. However, higher forming pressure requirements also entail higher equipment demands, leading to increased production costs. Therefore, the selection of forming pressure needs to be comprehensively considered based on various factors in the actual production process and determined according to the specific product’s added value to achieve an optimal balance between material performance and cost. The optimal formulation P4 obtained from the orthogonal experimental design can be considered an environmentally friendly, low-carbon material composition.

## 4. Conclusions

The present study investigated the performance of SSCM materials under static pressure processing, examining the influence of different forming pressures as well as varying dosages of MP and SSMP on material properties. The main conclusions are as follows.

Higher forming pressure can provide higher early strength, but the later strength of the material is mainly influenced by the hydration process. Compaction forming can significantly reduce porosity, with fewer harmful pores observed at higher pressures. Higher forming pressure can accelerate the early hydration reaction, but, in the later stages, the excessively high density and lower water–cement ratio may impede the progression of hydration reactions.

The optimal incorporation levels were determined to be 20% for MP and 30% for SSMP. For composite admixture, the highest compressive strength was achieved with 20% MP and 20% SSMP, reaching a material strength of 113.5 MPa. Even with 25% MP and 20% SSMP, there was still an improvement in strength compared to the control group, indicating the possibility of further admixture beyond a total admixture dosage of 45%.

When MP dosage, SSMP dosage, and forming pressure were simultaneously applied to the material, their influence decreased in the following order: SSMP dosage > forming pressure > MP dosage. The experimental results indicate that under static pressure processing, the optimal forming conditions for the specimens are 20% MP dosage, 20% SSMP dosage, and a forming pressure of 40 MPa. This configuration exhibits outstanding engineering ecological value, representing a green and clean concrete product that provides actionable guidance for developing low-carbon manufacturing solutions in practical production. However, excessive forming pressure may reduce the effective water-to-cement ratio and impede moisture migration, thereby reducing the degree of hydration. It can also damage the pore structure, causing original pores to be forcibly flattened rather than uniformly filled, resulting in irregular cracks that compromise long-term durability. In industrial applications, excessively high pressure will increase energy consumption while prolonging molding time. Therefore, in practical engineering applications, the choice of forming pressure should be determined based on the specific additional value of the product to achieve the optimal balance between material performance and cost.

## Figures and Tables

**Table 1:** Main properties of the SSCM.

Density (g/cm^3^)	Setting Time (min)		Compressive Strength (MPa)			Flexural Strength (MPa)		
2.83	Initial setting	Final setting	1 d	3 d	28 d	1 d	3 d	28 d
	25	42	36.5	45.3	66.2	6.2	6.7	7.6

**Table 2:** Chemical composition of raw materials (%).

Material	CaO	SiO_2_	Al_2_O_3_	Fe_2_O_3_	SO_3_	MgO	Loss on Ignition
SSCM	43.27	7.69	22.28	7.09	17.87	1.79	1.5
MP	48.23	17.75	5.43	28.03	0.38	0.19	0.9
SSMP	31.52	23.37	13.57	17.85	2.95	10.75	0.4

**Table 3:** Mixing proportions of the samples.

Series	Group	Forming Pressure (MPa)	SSCM (%)	MP (%)	SSMP (%)
I	FP20	20	100	0	0
	FP30	30	100	0	0
	FP40	40	100	0	0
II	M15	20	85	15	0
	M20	20	80	20	0
	M25	20	75	25	0
	S20	20	80	0	20
	S30	20	70	0	30
	S40	20	60	0	40
III	G1	20	65	15	20
	G2	20	55	15	30
	G3	20	45	15	40
	G4	20	60	20	20
	G5	20	50	20	30
	G6	20	40	20	40
	G7	20	55	25	20
	G8	20	45	25	30
	G9	20	35	25	40

**Table 4:** Factor levels of orthogonal experiments.

Levels	Factors		
	A (%)	B (%)	C (MPa)
1	15	1	15
2	20	2	20
3	25	3	25

**Table 5:** Response data collected from the orthogonal experiment.

Number	A	B	C	Compressive Strength (MPa)
P1	1	1	1	95.34
P2	1	2	2	105.43
P3	1	3	3	101.52
P4	2	1	2	119.85
P5	2	2	3	123.07
P6	2	3	1	60.25
P7	3	1	3	106.46
P8	3	2	1	74.95
P9	3	3	2	54.77
Mean A	100.76	107.21	76.85	95.34
Mean B	101.05	101.15	93.35	105.43
Mean C	78.73	72.18	110.35	101.52
Range	22.33	35.03	33.51	119.85

## Data Availability

The original contributions presented in this study are included in the article. Further inquiries can be directed to the corresponding author.

## Appendix A

**Table A1:** Porosity of materials under different pressures and casting processes.

	<10 nm	10–100 nm	100–1000 nm	1000–3000 nm	>3000 nm
Cast	3.38	2.7	9.19	1.82	14.94
FP20	2.95	5.34	7.50	1.00	2.17
FP40	2.22	5.64	8.10	0.51	1.79
